# Support for regulating smoking in private and public places by adults who currently smoke and recently quit smoking in Spain

**DOI:** 10.18332/tid/191797

**Published:** 2024-08-31

**Authors:** Marcela Fu, Yolanda Castellano, Olena Tigova, Mónica Pérez-Ríos, Pete Driezen, Susan C. Kaai, Anne C. K. Quah, Constantine I. Vardavas, Geoffrey T. Fong, Esteve Fernández

**Affiliations:** 1 Tobacco Control Unit, Catalan Institute of Oncology, WHO Collaborating Centre for Tobacco Control, L’Hospitalet de Llobregat, Barcelona, Spain; 2 Tobacco Control Research Group, Bellvitge Biomedical Research Institute, L’Hospitalet de Llobregat, Barcelona, Spain; 3 Department of Public Health, Mental Health, and Maternal and Child Health Nursing, Faculty of Nursing, University of Barcelona, Barcelona, Spain; 4 Centro de Investigación Biomédica en Red de Enfermedades Respiratorias, Instituto de Salud Carlos III, Madrid, España; 5 Department of Clinical Sciences, Faculty of Medicine and Health Sciences, University of Barcelona, Barcelona, Spain; 6 Department of Preventive Medicine and Public Health, University of Santiago de Compostela, Santiago de Compostela, Spain; 7 Centro de Investigación Biomédica en Red de Epidemiologií y Salud Pública, Instituto de Salud Carlos III, Madrid, España; 8 Department of Psychology, University of Waterloo, Waterloo, Canada; 9 School of Public Health Sciences, University of Waterloo, Waterloo, Canada; 10 School of Medicine, University of Crete, Heraklion, Greece; 11 European Network for Smoking and Tobacco Prevention, Brussels, Belgium; 12 Department of Oral Health Policy and Epidemiology, Harvard School of Dental Medicine, Harvard University, Boston, United States; 13 Ontario Institute for Cancer Research, Toronto, Canada

**Keywords:** outdoor places, private places, public places, smoke-free regulation, support

## Abstract

**INTRODUCTION:**

While indoor smoking restrictions are common, outdoor restrictions are still rare. We explored opinions and support for regulating smoking in different indoor and outdoor environments among adults who smoke and those who recently quit smoking, in Spain.

**METHODS:**

The 2021 ITC EUREST-PLUS Spain Survey is a cross-sectional study conducted among a nationally representative sample of 1006 adults aged ≥18 years who smoked cigarettes (n=867) or had recently quit smoking (n=139). Using Poisson regression with robust variance, we estimated adjusted prevalence and prevalence ratios of favorable opinions on regulating smoking in different indoor and outdoor environments and support for regulation in unregulated outdoor environments, by sociodemographic and smoking-related characteristics.

**RESULTS:**

There were highly favorable opinions for regulating smoking in places with minors (>95% in primary and secondary playgrounds, and cars with pre-school children and minors) and outdoor transportation (60–80%). There were less favorable opinions for regulating smoking in outdoor terraces of bars/pubs and restaurants (15–20%). Support for further total outdoor regulations on smoking was moderate for markets/shopping centers, public building entrances and swimming pools (40–60%), and low for restaurants/bars/pubs (29.2%). Having quit smoking, having no significant others who smoke and/or believing that cigarette smoke is harmful to others, were factors positively associated with favorable opinions and support for regulating smoking.

**CONCLUSIONS:**

The settings in Spain with the most favorable opinions for regulation among adults who smoke and have recently quit smoking are places with minors, private cars with others and outdoor areas of public transportation, while the settings with the least favorable opinions were outdoor terraces of bars, pubs, and restaurants. Support for further total outdoor smoking bans is generally moderate, but low for restaurants, bars, and pubs. Overall, these findings suggest the feasibility of extending smoke-free policies to other public and private settings to protect others from tobacco smoke exposure.

## INTRODUCTION

Exposure to secondhand smoke (SHS) is a recognized health hazard^1^. To address this preventable health risk, the World Health Organization (WHO) Framework Convention on Tobacco Control (FCTC) proposed comprehensive measures, including smoke-free policies^2^. These policies have been implemented in many countries with varying degrees of success. According to the Tobacco Control Scale, 75% of the 37 European countries assessed in 2021 scored >50% of the 22 available points for policies on bans/restrictions on smoking in workplaces, public places and private cars, but only 32% of the countries scored >90% of the points^3^. As only total bans comply with Article 8 of the WHO FCTC, further progress is needed to protect people from SHS exposure^1^.

Understanding the public’s views on smoking restrictions in public and private places is crucial to assessing their acceptability and future compliance, especially among those who smoke, who are directly affected by such restrictions. In this context, the International Tobacco Control Policy Evaluation (ITC) Project has been conducting prospective cohort studies in several countries since 2002 to assess the impact of the WHO FCTC on smoking behavior and attitudes^4^. In 2016, the EUREST-PLUS ITC 6 European Countries Survey included a cohort of adults who smoke from six European countries, including Spain^5^. This cohort was followed up in the six countries in 2018 and only in Spain in 2021^6^. Data from this latest survey provide the most up-to-date information available in Spain to assess opinions and attitudes towards regulating smoking in different settings, in the context of new regulations that have not yet been implemented as of June 2024^7^. The current Law 42/2010 represents a major step forward in the promotion of smoke-free environments, extending the partial regulations of the previous Law 28/2005 to all indoor public places without exception and introducing for the first time some restrictions in outdoor areas of healthcare centers, educational centers for minors, and playgrounds and recreational areas for children^8^. The Spanish health authorities are planning to extend the current smoke-free environments to other indoor and outdoor areas that have not yet been defined^7^.

Given this national scenario, we explored opinions and support for smoke-free policies in public and private settings, including some not covered by current legislation, among adults who smoke and those who recently quit smoking, in Spain.

## METHODS

### Study design

This is a cross-sectional study based on the 2021 ITC EUREST-PLUS Spain Survey, which is a follow-up survey of a nationally representative sample of adults aged ≥18 years who smoked at the time of recruitment in 2016 (Wave 1). Respondents were recontacted in 2018 (Wave 2) and 2021 (Wave 3). This analysis uses cross-sectional data from Wave 3. The original sample was randomly selected using a multistage design within geographical strata. Respondents interviewed in 2018 who agreed to be recontacted in the future were invited to participate in 2021. Respondents lost to follow-up were replaced with new participants selected from newly screened households using the same sampling frame. The final sample consisted of 1006 respondents who smoked or had quit smoking and provided valid information; 56.7% had been interviewed in the previous two waves. Further details of the methods can be found elsewhere^6^. The survey received ethical approval from the Research Ethics Boards of the Bellvitge University Hospital, Spain (PR248/17) and the University of Waterloo, Canada (REB#41105). All participants gave consent for participation.

### Measures

We asked participants for their opinions on smoke-free regulations in various indoor/outdoor places, some already regulated and some unregulated, and their support for total outdoor bans, most of which are not regulated by current law (Table 1).

**Table 1:** Regulation on smoking in different settings at national level in Spain at the time of the survey (2021)

*Setting*	*Regulation*
Schoolyards of primary and secondary schools	Banned
Children’s playgrounds	Banned
Outdoor campuses of health care centers	Banned
Terraces of bars, pubs, and restaurants	Partially banned
Public transport vehicles	Banned
Vehicles for commercial and service transport	Banned
Private vehicles	Unregulated
Outdoor areas of public transport, including stops	Unregulated
Entrances to public buildings	Unregulated
University campuses	Unregulated
Open sports facilities	Unregulated
Markets and shopping centers	Unregulated
Public urban parks	Unregulated
National parks	Unregulated
Beaches	Unregulated
Swimming pools	Unregulated

Source: Elaborated from Law 42/2010^8^.

*Opinions on smoke-free regulations in indoor/outdoor places*

These were assessed by asking: ‘At which of the following places do you think smoking should be allowed?’. Settings assessed were schoolyards of primary and secondary schools, outdoor terraces of of bars/pubs and restaurants, outdoor bus stops and subway/train stations, private cars (with pre-school children, with children aged <16 years, with others who do not smoke), within 5 m of public building entrances, beaches, and open stadiums for events. The response options were ‘yes’ or ‘no’. We describe ‘no’ responses as reflecting a favorable opinion on smoke-free regulation in a specific place. ‘Refused’ and ‘Don't know’ (RDK) responses were excluded (0.0–3.4% of responses) (Supplementary file Table S1).

*Support for further total outdoor smoking bans*

This was measured by the question: ‘Do you support or oppose a complete smoking ban in outdoor areas of the following places?’. Settings assessed were terraces of restaurants/bars/pubs, public buildings including entrances, markets/shopping centers, and swimming pools. The response options were ‘strongly support’, ‘support’, ‘oppose’, ‘strongly oppose’, recoded as support/oppose. We describe ‘support’ to reflect support for further outdoor smoking bans. RDK responses were excluded (2.0–4.4% of responses) (Supplementary file Table S1).

*Sociodemographic characteristics*

These were sex (male, female), age (<25, 25–39, 40–54, ≥55 years), education level (low: up to lower secondary education; medium: upper secondary to short-cycle tertiary education; high: completed university education), living with children aged <18 (yes/no), significant others who smoke [assuming that having a partner who smokes is more relevant than having friends who smoke, we categorized this variable as ‘a partner who smokes’ (regardless of having friends who smoke), ‘friends but not a partner who smokes’, and ‘no significant others who smoke’].

*Smoking-related characteristics*

These were smoking status (current smoking: having smoked ≥100 cigarettes in lifetime and currently smoking cigarettes at least less than monthly; former smoking: having quit since the two previous surveys)^9^; nicotine dependence, assessed with the Heaviness of Smoking Index^10^ (categorized as low: 0–2 points, moderate: 3–4 points, high: 5–6 points; for former smoking the score was 0); and having tried to quit in the last 18 months (yes/no).

*Belief about the harmfulness of SHS to others*

This was assessed with the statement: ‘Cigarette smoke is dangerous to non-smokers’. The response options were ‘strongly agree’, ‘agree’, ‘neither agree nor disagree’, ‘disagree’, ‘strongly disagree’, which were recoded as ‘agree’, ‘neither agree nor disagree’, ‘disagree’. RDK responses were excluded (1.3%) (Supplementary file Table S1).

### Analysis

We estimate the prevalence (with 95% confidence interval, CI) of favorable opinions on smoke-free regulations in different places and support for further outdoor smoking bans, stratifying by the independent variables. We used Poisson regression models with robust variance to estimate prevalence ratios (PR) and 95% CI for comparing opinions on smoke-free regulation and support for further outdoor smoking bans by all independent variables, adjusting for age, sex, and education level. All tests were two-tailed and statistical significance was set at p<0.05. All analyses used bootstrap replicate weights derived from the complex sampling design. We used Stata^®^ v.14 (Texas, USA) for all analyses.

## RESULTS

### Opinions on smoke-free regulation in indoor/outdoor places

**Figure 1:**
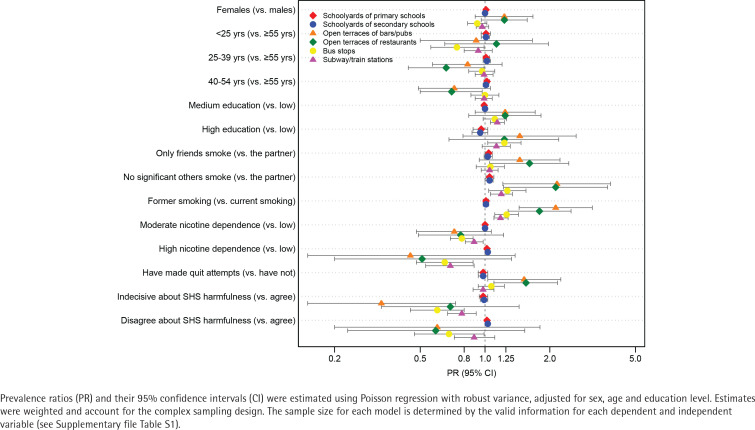
Factors associated with favorable opinions on smoke-free regulation in outdoor places with different regulation among a nationally representative sample of adults who smoke and recently quit smoking, ITC EUREST-PLUS Spain Survey, Spain, 2021 (N=1006)

Tables 2 and 3 show the prevalence of favorable opinions on smoke-free regulations in different settings. Among adults who smoke or recently quit, most think that smoking should not be allowed in schoolyards of primary (98.0%; 95% CI: 96.9–99.1) and secondary schools (97.4%; 95% CI: 96.2–98.6) (Table 2), with no differences according to the independent variables. High versus low nicotine dependence, and not believing versus believing that SHS is harmful to others were factors positively associated with this opinion in these settings (Figure 1) (and Supplementary file Table S2).

**Table 2:** Prevalence^a^ of favorable opinions on smoke-free regulation in outdoor places with different regulation among a nationally representative sample of adults who smoke and recently quit smoking, ITC EUREST-PLUS Spain Survey, Spain, 2021 (N=1006)

*Characteristics*	*Total*	*Schoolyards of primary schools*	*Schoolyards of secondary schools*	*Open terraces of bars/pubs*	*Open terraces of restaurants*	*Bus stops*	*Subway and train stations*
	*n*	*% (95% CI)*	*% (95% CI)*	*% (95% CI)*	*% (95% CI)*	*% (95% CI)*	*% (95% CI)*
**Total**	1006	98.0 (96.9–99.1)	97.4 (96.2–98.6)	15.2 (12.4–17.9)	18.4 (14.8–21.9)	62.3 (57.9–66.7)	78.3 (75.0–81.7)
**Sociodemographic characteristics**							
**Sex**							
Male	542	97.4 (95.9–98.9)	97.3 (95.8–98.8)	13.9 (10.5–17.2)	16.8 (13.2–20.5)	64.5 (59.2–69.8)	79.3 (75.3–83.2)
Female	464	98.7 (97.6–99.7)	97.6 (96.1–99.0)	16.5 (12.6–20.4)	20.0 (15.1–24.8)	60.1 (54.9–65.3)	77.4 (73.1–81.7)
**Age** (years)							
<25	68	97.60 (94.3–100)	97.1 (93.7–100)	16.7 (7.2–26.3)	26.2 (14.3–38.1)	49.3 (35.5–63.2)	76.3 (64.2–88.4)
25–39	272	98.0 (96.1–99.9)	98.2 (96.4–100)	15.1 (10.6–19.5)	15.1 (10.6–19.6)	63.1 (55.9–70.2)	79.9 (74.8–84.9)
40–54	360	99.1 (98.3–99.8)	97.7 (96.1–99.2)	12.9 (8.3–17.5)	15.8 (10.6–20.9)	63.6 (57.0–70.2)	78.2 (73.4–82.9)
≥55	306	97.2 (94.8–99.7)	96.8 (94.2–99.4)	16.9 (12.9–20.9)	21.3 (15.5–27.2)	63.4 (56.9–69.9)	77.8 (72.5–83.2)
**Education level**							
Low	506	98.6 (97.7–99.6)	97.9 (96.6–99.3)	13.5 (10.6–16.5)	16.7 (12.5–21.0)	59.3 (54.0–64.6)	73.7 (69.0–78.5)
Medium	391	98.2 (96.8–99.5)	98.0 (96.7–99.4)	16.4 (12.0–20.7)	20.3 (14.2–26.5)	63.8 (57.6–70.0)	83.3 (79.0–87.7)
High	109	94.5 (87.5–100)	93.0 (85.8–100)	18.9 (8.5–29.2)	19.4 (9.2–29.7)	72.0 (60.5–83.5)	83.0 (72.5–93.5)
**Children aged <18 years**							
Yes	331	99.4 (98.7–100)	99.1 (98.2–99.9)	13.0 (8.8–17.2)	16.0 (10.9–21.1)	62.0 (56.1–67.9)	79.0 (74.6–83.4)
No	675	97.4 (95.8–98.9)	96.7 (95.0–98.4)	16.2 (12.8–19.5)	19.5 (15.4–23.6)	62.5 (57.5–67.5)	78.0 (74.1–82.0)
**Significant others who smoke**							
Partner	287	96.0 (92.7–99.2)	95.6 (92.3–99.0)	10.9 (7.0–14.9)	12.2 (7.5–16.9)	58.7 (50.5–67.0)	74.8 (69.2–80.5)
Friends, but not the partner	620	98.7 (97.8–99.6)	98.0 (96.9–99.1)	15.9 (12.3–19.4)	20.1 (15.5–24.7)	62.1 (57.2–67.0)	78.6 (74.5–82.7)
Neither	98	100 (-)	99.5 (98.6–100)	24.0 (15.1–32.9)	26.6 (17.7–35.5)	75.6 (66.3–84.9)	88.5 (81.7–95.3)
**Smoking characteristics**							
**Smoking status**							
Current	867	97.9 (96.7–99.1)	97.3 (96.0–98.6)	12.9 (10.1–15.7)	16.3 (12.8–19.9)	59.9 (54.8–64.9)	76.2 (72.5–80.0)
Former	139	98.5 (95.9–100)	98.2 (95.6–100)	27.8 (19.2–36.5)	30.0 (21.5–38.5)	76.2 (68.9–83.4)	90.2 (85.0–95.5)
**Nicotine dependence**							
Low	450	98.2 (97.0–99.3)	97.5 (96.0–99.1)	15.6 (11.8–19.4)	19.4 (14.5–24.3)	65.9 (60.0–71.9)	81.2 (76.8–85.6)
Moderate	338	98.5 (97.4–99.7)	97.8 (96.2–99.3)	10.7 (7.1–14.3)	14.2 (8.0–20.3)	52.3 (46.0–58.6)	72.4 (66.2–78.5)
High	39	100 (-)	100 (-)	7.0 (0–14.4)	9.6 (1.1–18.1)	46.0 (31.0–61.1)	57.0 (41.5–72.4)
**Quit attempts** (last 18 months)							
Yes	132	95.9 (90.5–100)	95.3 (89.8–100)	18.5 (11.9–25.2)	23.3 (16.8–29.9)	63.8 (55.1–72.5)	75.1 (66.5–83.7)
No	735	98.3 (97.4–99.2)	97.7 (96.6–98.8)	11.9 (9.10–14.7)	15.1 (11.3–18.8)	59.2 (53.9–64.5)	76.5 (72.6–80.3)
**Belief about the harmfulness of SHS to others**							
Agree	833	98.1 (96.8–99.3)	97.4 (96.0–98.7)	16.8 (13.5–20.2)	19.5 (15.8–23.3)	66.7 (62.5–70.9)	80.7 (77.2–84.1)
Neither agree nor disagree	121	96.8 (93.0–100)	96.8 (92.9–100)	5.5 (1.4–9.5)	13.3 (2.9–23.8)	39.7 (28.1–51.3)	62.9 (53.5–72.3)
Disagree	39	100 (-)	100 (-)	9.9 (0–20.6)	12.1 (0.8–23.4)	44.6 (28.9–60.2)	71.7 (56.7–86.8)

SHS: secondhand smoke.

a Prevalence and 95% confidence intervals were computed using bootstrap replicate weights derived from the complex sampling design.

**Table 3:** Prevalence^a^ of favorable opinions on smoke-free regulation in outdoor places currently not regulated by law among a nationally representative sample of adults who smoke and recently quit smoking, ITC EUREST-PLUS Spain Survey, Spain, 2021 (N=1006)

*Characteristics*	*Total*	*Private cars with preschool children*	*Private cars with children aged <16 years*	*Private cars with others who do not smoke*	*Public building entrances*	*Beaches*	*Open stadiums*
	*n*	*% (95% CI)*	*% (95% CI)*	*% (95% CI)*	*% (95% CI)*	*% (95% CI)*	*% (95% CI)*
**Total**	1006	97.5 (96.4–98.7)	96.9 (95.7–98.1)	87.8 (84.5–91.1)	35.9 (31.1–40.7)	28.9 (24.7–33.0)	59.6 (54.8–64.3)
**Sociodemographic characteristics**							
**Sex**							
Male	542	97.2 (95.7–98.8)	97.2 (95.7–98.6)	86.4 (82.0–90.8)	36.7 (31.2–42.1)	30.2 (24.7–35.6)	56.7 (51.0–62.4)
Female	464	97.9 (96.7–99.0)	96.7 (95.0–98.3)	89.2 (85.8–92.6)	35.1 (29.7–40.5)	27.5 (22.8–32.2)	62.6 (57.1–68.1)
**Age** (years)							
<25	68	96.1 (92.4–99.8)	96.7 (93.2–100)	85.3 (76.6–94.0)	34.7 (20.2–49.2)	21.3 (11.7–30.9)	52.3 (38.5–66.1)
25–39	272	97.1 (95.0–99.3)	96.3 (93.5–99.1)	86.3 (81.0–91.7)	38.6 (32.3–44.8)	26.2 (20.9–31.6)	60.0 (52.8–67.3)
40–54	360	99.0 (98.2–99.8)	97.3 (95.8–98.9)	85.3 (80.4–90.2)	34.0 (27.5–40.4)	28.8 (22.9–34.7)	60.4 (54.2–66.6)
≥55	306	97.0 (94.6–99.3)	97.1 (94.7–99.4)	91.4 (87.5–95.3)	35.9 (29.0–42.8)	32.5 (26.0–39.1)	60.1 (52.4–67.8)
**Education level**							
Low	506	98.2 (97.1–99.2)	97.3 (95.9–98.7)	89.3 (85.7–92.9)	32.8 (27.2–38.5)	25.4 (20.5–30.3)	56.0 (49.5–62.4)
Medium	391	98.0 (96.8–99.3)	97.9 (96.5–99.3)	86.4 (81.9–90.8)	38.7 (32.9–44.5)	31.8 (26.4–37.2)	63.8 (57.5–70.2)
High	109	92.8 (85.8–99.8)	91.8 (84.7–99.0)	85.6 (75.1–96.0)	41.1 (29.7–52.5)	35.3 (23.7–46.9)	61.4 (49.5–73.3)
**Children aged <18 years**							
Yes	331	98.6 (97.6–99.7)	97.4 (95.5–99.3)	87.5 (83.0–91.9)	31.9 (25.7–38.1)	28.9 (23.2–34.6)	62.4 (55.4–69.5)
No	675	97.0 (95.5–98.6)	96.7 (95.1–98.3)	88.0 (84.3–91.6)	37.8 (32.5–43.0)	28.9 (24.0–33.7)	58.2 (52.8–63.5)
**Significant others who smoke**							
Partner	287	96.5 (93.4–99.5)	95.9 (92.7–99.1)	86.1 (80.9–91.3)	35.9 (28.7–43.2)	20.0 (13.8–26.2)	58.1 (49.7–66.5)
Friends, but not the partner	620	97.7 (96.5–98.9)	97.0 (95.7–98.2)	87.3 (83.3–91.3)	34.4 (28.8–40.0)	29.6 (24.3–34.9)	58.0 (52.5–63.6)
Neither	98	100 (-)	100 (-)	96.7 (93.2–100)	45.9 (35.7–56.2)	52.6 (43.2–62.0)	74.4 (65.8–83.0)
**Smoking characteristics**							
**Smoking status**							
Current	867	97.2 (96.0–98.5)	96.5 (95.1–97.9)	86.3 (82.5–90.1)	33.3 (28.0–38.6)	25.8 (20.9–30.6)	58.3 (53.1–63.4)
Former	139	99.3 (97.9–100)	99.2 (97.9–100)	96.1 (93.3–98.9)	50.6 (42.3–59.0)	46.1 (37.6–54.6)	66.8 (58.8–74.8)
**Nicotine dependence**							
Low	450	97.5 (96.1–98.9)	97.5 (96.1–98.9)	89.2 (85.7–92.8)	38.3 (32.1–44.5)	28.2 (22.6–33.7)	62.5 (56.3–68.6)
Moderate	338	97.9 (96.5–99.4)	96.2 (94.2–98.2)	83.8 (78.8–88.9)	28.3 (21.9–34.7)	21.9 (14.3–29.5)	54.2 (47.2–61.3)
High	39	97.9 (94.2–100)	96.9 (92.7–100)	85.1 (74.7–95.5)	23.4 (12.4–34.4)	27.8 (13.1–42.5)	40.9 (26.2–55.7)
**Quit attempts** (last 18 months)							
Yes	132	94.1 (88.5–99.7)	94.0 (88.4–99.7)	82.8 (70.8–94.8)	38.2 (29.4–47.1)	26.9 (18.3–35.5)	57.3 (48.0–66.5)
No	735	97.8 (96.7–98.9)	97.0 (95.7–98.2)	86.9 (83.3–90.6)	32.4 (26.8–38.0)	25.6 (20.5–30.7)	58.5 (53.2–63.8)
**Belief about the harmfulness of SHS to others**							
Agree	833	97.7 (96.4–98.9)	97.4 (96.1–98.7)	91.6 (88.7–94.5)	38.8 (33.8–43.8)	31.8 (27.1–36.6)	62.7 (57.9–67.6)
Neither agree nor disagree	121	97.2 (93.4–100)	94.8 (89.4–100)	70.3 (61.3–79.3)	24.6 (13.1–36.0)	13.0 (6.7–19.3)	47.0 (33.5–60.5)
Disagree	39	96.9 (91.7–100)	94.0 (87.3–100)	61.4 (44.3–78.6)	17.5 (3.2–31.8)	17.8 (6.4–29.2)	41.9 (26.5–57.4)

SHS: secondhand smoke.

a Prevalence and 95% confidence intervals were computed using bootstrap replicate weights derived from the complex sampling design.

A favorable opinion on smoke-free regulation was less prevalent for outdoor transportation (bus stops: 62.3%; 95% CI: 57.9–66.7; subways/train stations: 78.3%; 95% CI: 75.0–81.7) (Table 2). Those who formerly smoked, have low nicotine dependence, and believe that SHS is harmful to others are more likely to agree with smoke-free regulations in these settings. As shown in Figure 1, factors positively associated with this opinion were moderate/high (vs low) education level, not having significant others who smoke (vs having a partner who smokes) and former (vs current) smoking. In contrast, factors negatively associated were moderate/high nicotine dependence and neither agreeing nor disagreeing with the statement that SHS is harmful to others (Figure 1) (and Supplementary file Table S2).

Conversely, few adults who smoke or recently quit have favorable opinions on smoke-free regulation for outdoor terraces of bars/pubs (15.2%) and restaurants (18.4%); this is higher among those who have quit smoking (27.8% and 30.0%, respectively) (Table 2). Factors positively associated with this opinion were having no significant others who smoke, having previously smoked, and having recently tried to quit smoking (Figure 1) (and Supplementary file Table S2).

**Figure 2:**
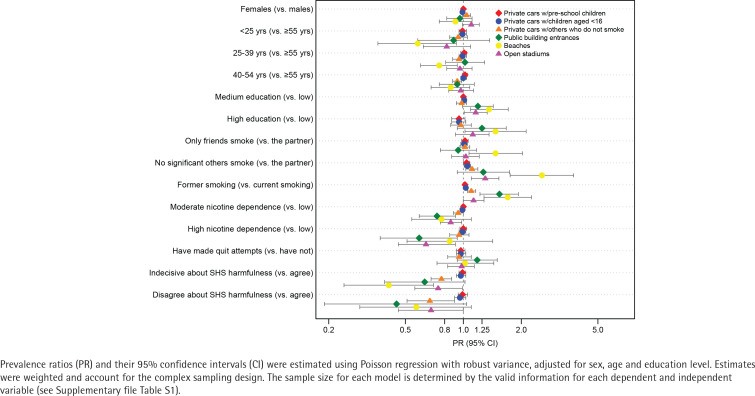
Factors associated with favorable opinions on smoke-free regulation in outdoor places currently not regulated by law among a nationally representative sample of adults who smoke and recently quit smoking, ITC EUREST-PLUS Spain Survey, Spain, 2021 (N=1006)

Another setting for which adults who smoke or recently quit are most in favor of smoking restrictions was in private cars with: pre-school children (97.5%; 95% CI: 96.4–98.7), children aged <16 years (96.9%; 95% CI: 95.7–98.1), and others who do not smoke (87.8%; 95% CI: 84.5–91.1) (Table 3). Favorable opinions about smoking restrictions in a car with others who do not smoke were more frequent among those without significant others who smoke, who had previously smoked, and believing that SHS is harmful to others (all >90%). Having no significant others who smoke and having previously smoked were more positively associated with support for such a regulation, whereas being aged 40–54 years (vs the oldest age group) and disagreeing and neither agreeing nor disagreeing that SHS is harmful to others were negatively associated with such support (Figure 2) (and Supplementary file Table S3).

Around 30–60% of adults who smoke or recently quit are in favor of smoke-free regulations on beaches, at public building entrances and in open-air stadiums, particularly those with no significant others who smoked, have previously smoked, and believe that others are adversely affected by SHS (Table 3). Factors positively associated with this opinion were moderate education level, the absence of a significant other who smokes, and having previously smoked. Conversely, moderate/high nicotine dependence and neither agreeing nor disagreeing with the statement that SHS is harmful to others were more negatively associated with this opinion (Figure 2) (and Supplementary file Table S3).

### Support for further total outdoor smoking bans

**Figure 3:**
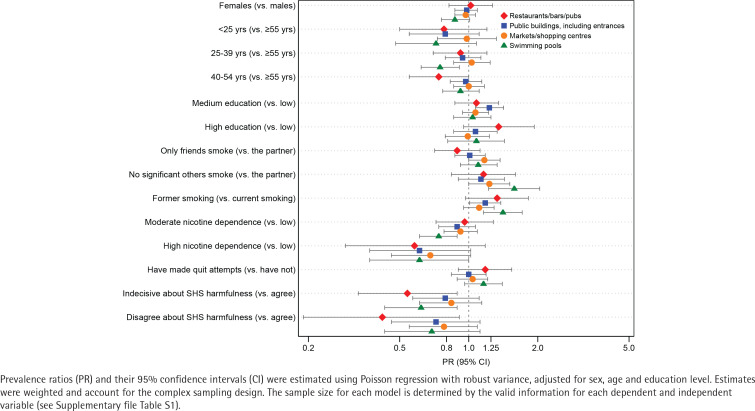
Factors associated with support for further total outdoor smoking bans among a nationally representative sample of adults who smoke and recently quit smoking, ITC EUREST-PLUS Spain Survey, Spain, 2021 (N=1006)

Less than 60% of adults who smoke or recently quit support further complete smoking bans in outdoor environments (Table 4), including markets/shopping centers (57.2%; 95% CI: 51.7–62.6), public building entrances (50.9%; 95% CI: 46.2–55.5), and swimming pools (43.4%; 95% CI: 39.2–47.7). In this last setting, the highest levels of support come from older participants (48.9%; 95% CI: 42.5–55.3), with no significant others who smoke (62.9%; 95% CI: 52.5–73.3) and believing that SHS is harmful to others (46.5%; 95% CI: 41.8–51.3) (Table 4). Support for smoke-free swimming pools was positively associated with having no significant others who smoke and having previously smoked; negatively associated factors included being aged 25–39 years, moderate nicotine dependence, and not agreeing or disagreeing with the statement that SHS is harmful to others. Only moderate education level and having previously smoked were positively associated with support for public building entrances (Figure 3) (and Supplementary file Table S4).

**Table 4:** Prevalence^a^ of support for further total outdoor smoking bans among a nationally representative sample of adults who smoke and recently quit smoking, ITC EUREST-PLUS Spain Survey, Spain, 2021 (N=1006)

*Characteristics*	*Total*	*Restaurants, bars, and pubs*	*Public buildings, including entrances*	*Markets and shopping centers*	*Swimming pools*
	*n*	*% (95% CI)*	*% (95% CI)*	*% (95% CI)*	*% (95% CI)*
**Total**	1006	29.2 (24.9–33.6)	50.9 (46.2–55.5)	57.2 (51.7–62.6)	43.4 (39.2–47.7)
**Sociodemographic characteristics**					
**Sex**					
Male	542	29.3 (23.7–34.9)	51.1 (45.7–56.5)	57.9 (51.5–64.4)	46.4 (41.0–51.9)
Female	464	29.2 (24.1–34.2)	50.6 (45.2–56.0)	56.4 (50.6–62.2)	40.4 (35.3–45.4)
**Age** (years)					
<25	68	26.3 (14.9–37.8)	44.1 (30.0–58.1)	56.7 (41.4–72.1)	35.7 (21.7–49.6)
25–39	272	31.1 (24.5–37.6)	51.0 (44.2–57.7)	58.7 (51.3–66.2)	36.8 (31.2–42.4)
40–54	360	24.6 (17.9–31.3)	51.6 (45.3–57.8)	56.9 (49.6–64.1)	44.6 (37.9–51.2)
≥55	306	32.5 (26.6–38.4)	51.7 (44.5–58.8)	56.4 (48.7–64.1)	48.9 (42.5–55.3)
**Education level**					
Low	506	27.7 (22.9–32.6)	47.1 (41.4–52.8)	55.8 (49.4–62.1)	43.8 (37.9–49.7)
Medium	391	29.1 (23.0–35.3)	56.4 (50.3–62.4)	59.6 (52.9–66.4)	42.5 (36.5–48.5)
High	109	36.8 (25.9–47.8)	49.5 (39.8–59.1)	55.3 (44.3–66.4)	45.0 (33.5–56.5)
**Children aged <18 years**					
Yes	331	29.0 (21.5–36.5)	53.8 (47.0–60.6)	58.2 (51.0–65.4)	43.3 (37.4–49.2)
No	675	29.4 (25.1–33.6)	49.5 (44.5–54.5)	56.7 (50.8–62.7)	43.5 (38.6–48.4)
**Significant others who smoke**					
Partner	287	29.8 (23.4–36.2)	50.0 (42.7–57.3)	51.1 (43.3–58.9)	38.1 (30.6–45.6)
Friends, but not the partner	620	27.7 (22.8–32.7)	50.3 (44.9–55.8)	59.3 (52.9–65.7)	43.1 (38.6–47.7)
Neither	98	37.2 (26.5–47.8)	56.7 (46.3–67.1)	62.2 (52.0–72.4)	62.9 (52.5–73.3)
**Smoking characteristics**					
**Smoking status**					
Current smoking	867	27.1 (21.8–32.3)	48.9 (43.7–54.0)	55.7 (49.7–61.7)	40.3 (35.3–45.2)
Former smoking	139	42.2 (24.7–59.8)	61.7 (42.9–80.5)	70.4 (55.2–85.7)	46.5 (25.5–67.4)
**Nicotine dependence**					
Low	450	28.4 (22.4–34.3)	51.6 (45.6–57.6)	58.3 (51.1–65.6)	45.3 (38.8–51.7)
Moderate	338	26.7 (19.4–33.9)	47.1 (39.4–54.8)	54.1 (46.2–62.1)	34.5 (28.2–40.7)
High	39	17.2 (5.3–29.1)	33.8 (16.4–51.1)	40.6 (24.5–56.7)	31.6 (16.5–46.8)
**Quit attempts** (last 18 months)					
Yes	132	32.5 (25.0–40.1)	49.0 (39.9–58.2)	58.2 (49.2–67.2)	45.9 (38.3–53.5)
No	735	26.8 (21.3–32.3)	49.5 (44.3–54.7)	56.0 (49.8–62.2)	39.6 (34.2–45.0)
**Belief about the harmfulness of SHS to others**					
Agree	833	31.9 (27.0–36.9)	53.0 (48.2–57.8)	59.1 (53.6–64.6)	46.5 (41.8–51.3)
Neither agree nor disagree	121	17.3 (9.1–25.6)	42.4 (28.7–56.2)	49.6 (34.5–64.7)	28.2 (18.4–38.0)
Disagree	39	13.1 (3.5–22.6)	37.6 (21.2–54.1)	46.0 (30.3–61.7)	32.5 (16.8–48.1)

SHS: secondhand smoke.

a Prevalence and 95% confidence intervals were computed using bootstrap replicate weights derived from the complex sampling design.

Restaurants/bars/pubs were found to have the lowest level of support for further total outdoor smoking bans (29.2%; 95% CI: 24.9–33.6) (Table 4). The only factor negatively associated with such support was disagreeing or neither agreeing nor disagreeing with the statement that SHS is harmful to others (Figure 3) (and Supplementary file Table S4).

## DISCUSSION

Depending on the settings and the regulations in place, there were mixed opinions and support for smoke-free regulations by adults who smoke and recently quit smoking. Opinions on indoor/outdoor smoke-free regulation ranged from 98.0% for schoolyards of primary schools to 15.2% for outdoor bars/pub terraces, while support for further outdoor smoking bans ranged from 57.2% for markets/shopping centers to 29.2% for restaurants/bars/pubs. There are several possible explanations for this wide range of opinions and support.

### Opinions on smoke-free regulation in indoor/outdoor places

Given that smoking is already banned in schoolyards of primary and secondary schools, and that these are places where minors are present, the opinion on smoke-free regulations in these settings is highly favorable. The same occurs for cars with minors, and slightly less for cars with adults who do not smoke, although private cars are not covered by the current legislation (Table 1). Compared to the 2016 survey, favorable opinions in these settings increased slightly since then (i.e. >90%)^11^. This may reflect the public’s awareness of the health effects of SHS exposure on vulnerable populations, such as children^12^. It may also be an indicator of their readiness to introduce regulations on smoking in private cars with vulnerable groups such as children and pregnant women, both circumstances in which smoking may be banned under further legislation, as in other European countries^3^. In some countries, the prevalence of voluntary smoke-free rules in private cars with children was as high as 65% in 2010–2011, consistent with the widely expressed support (>80%) for banning smoking in cars with children^11,13^.

Spanish tobacco Law 28/2005 regulated smoking on public transportation, but only indoors. Although moderate proportions of adults who smoke are in favor of not allowing smoking in outdoor areas, we observed important increases compared to opinions expressed in 2016, by 30 percentage points for bus stops and 15 percentage points for subway/train stations^11^, suggesting the feasibility of further regulation in these settings. Outdoor terraces of restaurants/bars/pubs receive, however, less favorable opinions. This was not surprising, because a weak regulation was initially established for the indoor areas of these venues, which was amended (by Law 42/2010) to a complete ban on smoking indoors and a partial ban outdoors, affecting those terraces with a roof and more than two walls or faces^8,14^. While favorable opinions increased from 3.4% to 15.2% for bar/pub terraces and from 4.2% to 18.4% for restaurant terraces between 2016 and 2021, they remain relatively low and consistent with observed low compliance^15^. This may be because people usually perceive SHS to dissipate quickly and the potential for exposure is low. However, there is evidence that SHS exposure can be as high as in indoor smoking areas^16^. Therefore, further regulation is expected to include these settings. The growing support for regulation of these settings in our study and others^17^, suggests that it can be implemented. In fact, smoking in these settings was already regulated during the COVID-19 pandemic in Spain^18,19^, based on WHO recommendations in response to the pandemic^20^, although this was a temporary measure.

Another setting where SHS exposure is typically underestimated is access to public buildings. Our data show that only 36% of adults who currently smoke or recently quit favor regulating smoking in this setting, but this prevalence is higher than that in 2016 (20.5%). Although some studies have demonstrated the potential for outdoor smoke to drift indoors^16,21^, smoking bans in building entrances are still rare, having been implemented in only a few jurisdictions^22,23^. The same is true for beaches and stadiums, although there is increasing favorable opinion on regulations in these settings^24^. In Spain, voluntary regulations for beaches are increasing^25^. In Barcelona, a city council intervention that included a smoking ban on beaches was well accepted and effective in reducing smoking and the visibility of people smoking^26^.

### Support for further total outdoor smoking bans

Support for complete smoking bans in outdoor places is generally moderate (40–60% for public buildings entrances, markets/shopping centers, and swimming pools), which is consistent with evidence showing high support (69%) for smoke-free outdoor non-hospitality settings (playgrounds, streets, beaches), with no difference between countries with and without existing regulations^27^. However, total outdoor bans in restaurants/bars/pubs, which are partially regulated in Spain, had the lowest support, probably due to strong lobbying from the hospitality sector since the enactment of the Law 28/2005^28^, which predicted negative economic consequences for the hospitality sector that never materialized^29^. Cultural reasons may also be linked to low support in these settings. The terraces are places where people socialize, so cultural changes are hard to achieve. Although low support in these settings among adults who smoke has also been found in other countries without regulation^30,31^, it increased after ban implementation^32-34^.

Our data indicate relevant factors associated with opinion and support for smoke-free outdoor environments, which are particularly strong among those who quit smoking, without significant others who smoke, and believing that SHS is harmful to others. These findings highlight the importance of promoting not only cessation but also educational campaigns focusing on the health effects of SHS exposure. In Spain, there are several local initiatives to ban smoking in outdoor settings, such as beaches, stadiums, and bus stops, with good acceptance^25^. It therefore seems feasible to include smoke-free outdoor areas in national legislation^35^. Nevertheless, it is crucial to raise awareness of SHS exposure in outdoor environments, especially on the terraces of restaurants, bars, and pubs, where exposure can be high^15^.

### Strengths and limitations

The main strength of this research is its robust survey design, which allowed us to study a nationally representative sample of adults who smoke and recently quit smoking in Spain. The use of the same questionnaire as in previous surveys allowed us to assess the reliability of the results; the current survey included additional settings that will be useful to explore changes in opinion and support for further regulation in the near future, examining several socio-economic and smoking-related variables.

A limitation of this study is the cross-sectional nature of the analysis, which precludes any causal relationship between the variables examined. In addition, responses may be subject to information bias, although the results are consistent with the previous survey^11^, with higher favorable opinions and support for smoke-free policies in all settings. Also, we did not adjust the analyses for wave of recruitment to account for the number of times participants had previously responded to the survey, nor did we stratify for this variable to assess effect modification; therefore, we cannot disregard some overestimation of favorable opinions and support. Finally, although we used a common questionnaire used in other ITC surveys, our results are not necessarily generalizable to other countries.

## CONCLUSIONS

Opinions and support in Spain for smoke-free regulations in different settings were heterogeneous among adults who smoke and those who recently quit smoking, depending on the setting assessed and the current regulation in place. Settings receiving the most favorable opinions to be regulated were places where minors are present, private cars with others, and outdoor areas of public transportation, while the least favorable opinions were expressed for outdoor terraces of bars, pubs, and restaurants. Support for further total outdoor smoking bans is generally moderate, but low for restaurants, bars, and pubs, which are partially regulated. Overall, these results suggest that smoke-free policies could be extended to other public and private settings to protect others from exposure to SHS. In Spain, legislation for smoke-free outdoor environments is on the horizon^36^, so there is a need for educational campaigns to raise awareness of SHS, especially in outdoor settings.

## Supplementary Material



## Data Availability

The data supporting this research are available from the authors on reasonable request.
