# Supplementary material for: Support for regulating smoking in private and public places by adults who currently smoke and recently quit smoking in Spain
Source: Tob Induc Dis. 2024 Aug 31;22:10.18332/tid/191797. doi: 10.18332/tid/191797 (PMC11365038; doi:10.18332/tid/191797)
Supplement: Supplementary file 1 [file TID-22-149-s1.pdf]

Supplementary Table S1. Valid information and missing values for the variables analysed. ITC EUREST-PLUS Spain Survey, Spain, 2021 (n=1006).

Variables	Valid n	Missing values			
		Refused	Don't know	Total	
		n	n	n	%
**Dependent variables**					
Opinions on smoke-free regulation in:					
Schoolyards of primary schools	1006	0	0	0	0.0
Schoolyards of secondary schools	1005	0	1	1	0.1
Open terraces of bars/pubs	1000	1	5	6	0.6
Open terraces of restaurants	1000	1	5	6	0.6
Outdoor bus stops	994	1	11	12	1.2
Outdoor subway and train stations	972	1	33	34	3.4
Private cars with pre-school children	1000	0	6	6	0.6
Private cars with children aged <16	995	0	11	11	1.1
Private cars with others who do not smoke	983	0	23	23	2.3
Within 5 m of public building entrances	1001	1	4	5	0.5
Beaches	988	1	17	18	1.8
Open stadiums for events	983	1	22	23	2.3
Support for further total outdoor smoking bans in:					
Restaurants, bars and pubs	986	5	15	20	2.0
Public buildings, including entrances	982	5	19	24	2.4
Markets and shopping centres	983	3	20	23	2.3
Swimming pools	962	3	41	44	4.4
**Independent variables**					
Sociodemographics					
Sex	1006	0	0	0	0.0
Age	1006	0	0	0	0.0
Educational level	1006	0	0	0	0.0
Children aged <18	1006	0	0	0	0.0
Significant others who smoke	1005	0	1	1	0.1
Smoking characteristics					
Smoking status	1006	0	0	0	0.0
Nicotine dependence	1006	0	0	0	0.0
Quit attempts (last 18 months)	1006	0	0	0	0.0
Belief about the harmfulness of SHS to others	993	1	12	13	1.3

SHS: second-hand smoke.

Supplementary Table S2. Factors associated with favorable opinions on smoke-free regulation in outdoor places with different regulation among a nationally representative sample of adults who smoke and recently quit smoking. ITC EUREST-PLUS Spain Survey, Spain, 2021 (n=1006).

Characteristics	n	Schoolyards of primary schools	Schoolyards of secondary schools	Open terraces of bars/pubs	Open terraces of restaurants	Bus stops	Subway and train stations
		PR[a] (95% CI)	PR[a] (95% CI)	PR[a] (95% CI)	PR[a] (95% CI)	PR[a] (95% CI)	PR[a] (95% CI)
**Sociodemographic characteristics**							
Sex							
Male	542	Ref.	Ref.	Ref.	Ref.	Ref.	Ref.
Female	464	1.01 (1.00-1.03)	1.00 (0.98-1.02)	1.23 (0.90-1.67)	1.23 (0.96-1.57)	0.92 (0.83-1.02)	0.97 (0.91-1.04)
Age (years)							
<25	68	1.01 (0.96-1.06)	1.01 (0.96-1.05)	0.91 (0.50-1.66)	1.13 (0.65-1.97)	0.74 (0.56-0.99)	0.93 (0.80-1.08)
25-39	272	1.01 (0.98-1.05)	1.02 (0.98-1.06)	0.83 (0.57-1.20)	0.66 (0.44-0.99)	0.97 (0.84-1.11)	0.99 (0.90-1.09)
40-54	360	1.02 (0.99-1.05)	1.01 (0.98-1.04)	0.72 (0.49-1.06)	0.70 (0.50-0.97)	1.00 (0.86-1.16)	0.99 (0.90-1.08)
≥55	306	Ref.	Ref.	Ref.	Ref.	Ref.	Ref.
Educational level							
Low	506	Ref.	Ref.	Ref.	Ref.	Ref.	Ref.
Medium	391	0.99 (0.97-1.01)	1.00 (0.98-1.02)	1.24 (0.90-1.71)	1.24 (0.84-1.82)	1.11 (0.98-1.26)	1.14 (1.06-1.23)
High	109	0.96 (0.88-1.03)	0.95 (0.87-1.03)	1.45 (0.79-2.65)	1.23 (0.68-2.20)	1.23 (1.03-1.47)	1.13 (0.97-1.31)
Children aged <18							
Yes	331	1.01 (1.00-1.03)	1.02 (1.00-1.04)	0.85 (0.58-1.25)	0.90 (0.62-1.30)	1.00 (0.91-1.11)	1.02 (0.95-1.09)
No	675	Ref.	Ref.	Ref.	Ref.	Ref.	Ref.
Significant others who smoke							
Partner	287	Ref.	Ref.	Ref.	Ref.	Ref.	Ref.
Friends, but not the partner	620	1.04 (1.00-1.08)	1.03 (0.99-1.08)	1.45 (0.94-2.23)	1.61 (1.05-2.45)	1.06 (0.91-1.23)	1.05 (0.96-1.15)
Neither	98	1.05 (1.01-1.10)	1.05 (1.00-1.09)	2.16 (1.21-3.83)	2.13 (1.22-3.71)	1.27 (1.04-1.55)	1.19 (1.06-1.34)

Characteristics	n	Schoolyards of primary schools	Schoolyards of secondary schools	Open terraces of bars/pubs	Open terraces of restaurants	Bus stops	Subway and train stations
		PR[a] (95% CI)	PR[a] (95% CI)	PR[a] (95% CI)	PR[a] (95% CI)	PR[a] (95% CI)	PR[a] (95% CI)
**Smoking characteristics**							
Smoking status							
Current smoking	867	Ref.	Ref.	Ref.	Ref.	Ref.	Ref.
Former smoking	139	1.01 (0.98-1.04)	1.01 (0.98-1.04)	2.13 (1.44-3.15)	1.79 (1.28-2.51)	1.26 (1.11-1.43)	1.18 (1.10-1.28)
Nicotine dependence							
Low	450	Ref.	Ref.	Ref.	Ref.	Ref.	Ref.
Moderate	338	1.00 (0.99-1.02)	1.00 (0.98-1.02)	0.72 (0.48-1.07)	0.77 (0.49-1.22)	0.78 (0.69-0.88)	0.89 (0.81-0.98)
High	39	1.02 (1.01-1.03)	1.03 (1.01-1.04)	0.45 (0.15-1.38)	0.51 (0.20-1.33)	0.65 (0.48-0.88)	0.69 (0.53-0.89)
Quit attempts (last 18 months)							
Yes	132	0.98 (0.93-1.03)	0.98 (0.93-1.03)	1.52 (1.03-2.25)	1.55 (1.10-2.17)	1.07 (0.93-1.23)	0.98 (0.88-1.10)
No	735	Ref.	Ref.	Ref.	Ref.	Ref.	Ref.
Belief about the harmfulness of SHS to others							
Agree	833	Ref.	Ref.	Ref.	Ref.	Ref.	Ref.
Neither agree nor disagree	121	0.98 (0.94-1.03)	0.99 (0.95-1.03)	0.33 (0.15-0.73)	0.69 (0.33-1.44)	0.60 (0.45-0.80)	0.78 (0.67-0.91)
Disagree	39	1.02 (1.01-1.04)	1.03 (1.01-1.04)	0.60 (0.20-1.80)	0.59 (0.23-1.53)	0.68 (0.47-0.99)	0.89 (0.72-1.11)

CI: confidence interval; PR: prevalence ratio; SHS: second-hand smoke.

[a]Prevalence ratios and 95% confidence intervals were estimated using Poisson regression models with robust variance adjusted for age, sex, and educational level.

Supplementary Table S3. Factors associated with favorable opinions on smoke-free regulation in outdoor places currently not regulated by law among a nationally representative sample of adults who smoke and recently quit smoking. ITC EUREST-PLUS Spain Survey, Spain, 2021 (n=1006).

Characteristics	n	Private cars with pre-school children	Private cars with children aged <16	Private cars with others who do not smoke	Public building entrances	Beaches	Open stadiums
		PR[a] (95% CI)	PR[a] (95% CI)	PR[a] (95% CI)	PR[a] (95% CI)	PR[a] (95% CI)	PR[a] (95% CI)
**Sociodemographic characteristics**							
Sex							
Male	542	Ref.	Ref.	Ref.	Ref.	Ref.	Ref.
Female	464	1.00 (0.99-1.02)	0.99 (0.97-1.01)	1.04 (0.99-1.09)	0.96 (0.82-1.11)	0.91 (0.75-1.11)	1.10 (0.99-1.21)
Age (years)							
<25	68	0.99 (0.95-1.04)	0.99 (0.95-1.04)	0.94 (0.85-1.05)	0.89 (0.58-1.37)	0.58 (0.36-0.92)	0.82 (0.62-1.09)
25-39	272	1.01 (0.97-1.04)	0.99 (0.95-1.04)	0.95 (0.88-1.02)	1.02 (0.81-1.29)	0.75 (0.60-0.93)	0.96 (0.82-1.11)
40-54	360	1.02 (0.99-1.05)	1.00 (0.97-1.04)	0.93 (0.88-0.98)	0.93 (0.75-1.14)	0.86 (0.68-1.08)	0.97 (0.84-1.13)
≥55	306	Ref.	Ref.	Ref.	Ref.	Ref.	Ref.
Educational level							
Low	506	Ref.	Ref.	Ref.	Ref.	Ref.	Ref.
Medium	391	1.00 (0.98-1.01)	1.01 (0.99-1.03)	0.98 (0.92-1.04)	1.19 (0.99-1.43)	1.36 (1.09-1.71)	1.16 (1.01-1.33)
High	109	0.95 (0.87-1.02)	0.95 (0.87-1.03)	0.97 (0.86-1.10)	1.25 (0.93-1.67)	1.47 (1.02-2.12)	1.12 (0.91-1.36)
Children aged <18							
Yes	331	1.01 (0.99-1.03)	1.01 (0.98-1.03)	1.01 (0.95-1.08)	0.84 (0.68-1.05)	1.08 (0.85-1.38)	1.07 (0.94-1.22)
No	675	Ref.	Ref.	Ref.	Ref.	Ref.	Ref.
Significant others who smoke							
Partner	287	Ref.	Ref.	Ref.	Ref.	Ref.	Ref.
Friends, but not the partner	620	1.02 (0.98-1.06)	1.01 (0.97-1.05)	1.02 (0.96-1.08)	0.94 (0.76-1.17)	1.47 (1.07-2.03)	1.03 (0.87-1.21)
Neither	98	1.04 (1.00-1.08)	1.05 (1.01-1.09)	1.11 (1.03-1.19)	1.27 (0.93-1.74)	2.56 (1.75-3.74)	1.30 (1.10-1.53)
**Smoking characteristics**							
Smoking status							
Current smoking	867	Ref.	Ref.	Ref.	Ref.	Ref.	Ref.
Former smoking	139	1.02 (1.00-1.04)	1.03 (1.01-1.05)	1.10 (1.05-1.16)	1.54 (1.22-1.93)	1.70 (1.28-2.26)	1.13 (1.00-1.28)

Characteristics	n	Private cars with pre-school children	Private cars with children aged <16	Private cars with others who do not smoke	Public building entrances	Beaches	Open stadiums
		PR[a] (95% CI)	PR[a] (95% CI)	PR[a] (95% CI)	PR[a] (95% CI)	PR[a] (95% CI)	PR[a] (95% CI)
Nicotine dependence							
Low	450	Ref.	Ref.	Ref.	Ref.	Ref.	Ref.
Moderate	338	1.00 (0.98-1.02)	0.99 (0.96-1.01)	0.94 (0.89-1.00)	0.73 (0.59-0.91)	0.77 (0.54-1.10)	0.86 (0.76-0.98)
High	39	1.00 (0.96-1.04)	0.99 (0.95-1.03)	0.95 (0.85-1.07)	0.59 (0.37-0.93)	0.85 (0.51-1.42)	0.64 (0.46-0.91)
Quit attempts (last 18 months)							
Yes	132	0.97 (0.92-1.02)	0.97 (0.92-1.03)	0.95 (0.83-1.10)	1.18 (0.93-1.50)	1.02 (0.73-1.44)	0.98 (0.83-1.14)
No	735	Ref.	Ref.	Ref.	Ref.	Ref.	Ref.
Belief about the harmfulness of SHS to others							
Agree	833	Ref.	Ref.	Ref.	Ref.	Ref.	Ref.
Neither agree nor disagree	121	0.99 (0.95-1.03)	0.97 (0.92-1.03)	0.77 (0.68-0.87)	0.63 (0.39-1.02)	0.41 (0.24-0.70)	0.74 (0.56-0.99)
Disagree	39	0.99 (0.94-1.05)	0.96 (0.90-1.03)	0.67 (0.51-0.90)	0.45 (0.19-1.04)	0.57 (0.29-1.10)	0.68 (0.46-1.00)

CI: confidence interval; PR: prevalence ratio; SHS: second-hand smoke.

[a]Prevalence ratios and 95% confidence intervals were estimated using Poisson regression models with robust variance adjusted for age, sex, and educational level.

Supplementary Table S4. Factors associated with support for further total outdoor smoking bans among a nationally representative sample of adults who smoke and recently quit smoking. ITC EUREST-PLUS Spain Survey, Spain, 2021 (n=1006).

Characteristics		Restaurants, bars, and pubs	Public buildings, including entrances	Markets and shopping centres	Swimming pools
	n	PR[a] (95% CI)	PR[a] (95% CI)	PR[a] (95% CI)	PR[a] (95% CI)
**Sociodemographic characteristics**					
Sex					
Male	542	Ref.	Ref.	Ref.	Ref.
Female	464	1.02 (0.82-1.27)	0.98 (0.87-1.09)	0.97 (0.87-1.07)	0.87 (0.76-1.01)
Age (years)					
<25	68	0.78 (0.50-1.20)	0.79 (0.55-1.11)	0.98 (0.73-1.32)	0.72 (0.48-1.08)
25-39	272	0.92 (0.70-1.20)	0.94 (0.79-1.13)	1.03 (0.86-1.24)	0.75 (0.62-0.91)
40-54	360	0.74 (0.55-1.00)	0.97 (0.83-1.14)	1.00 (0.86-1.17)	0.92 (0.77-1.11)
≥55	306	Ref.	Ref.	Ref.	Ref.
Educational level					
Low	506	Ref.	Ref.	Ref.	Ref.
Medium	391	1.08 (0.87-1.35)	1.23 (1.07-1.42)	1.07 (0.94-1.22)	1.04 (0.86-1.25)
High	109	1.35 (0.95-1.93)	1.07 (0.86-1.33)	0.99 (0.79-1.23)	1.08 (0.81-1.43)
Children aged <18					
Yes	331	1.09 (0.84-1.41)	1.10 (0.95-1.27)	1.02 (0.90-1.17)	1.07 (0.92-1.26)
No	675	Ref.	Ref.	Ref.	Ref.
Significant others who smoke					
Partner	287	Ref.	Ref.	Ref.	Ref.
Friends, but not the partner	620	0.89 (0.71-1.12)	1.01 (0.87-1.18)	1.17 (1.00-1.37)	1.10 (0.92-1.33)
Neither	98	1.16 (0.84-1.60)	1.13 (0.90-1.43)	1.23 (1.00-1.51)	1.58 (1.22-2.04)
**Smoking characteristics**					
Smoking status					
Current smoking	867	Ref.	Ref.	Ref.	Ref.
Former smoking	139	1.33 (0.97-1.82)	1.18 (1.01-1.38)	1.11 (0.95-1.29)	1.41 (1.16-1.71)

Characteristics	n	Restaurants, bars, and pubs	Public buildings, including entrances	Markets and shopping centres	Swimming pools
		PR[a] (95% CI)	PR[a] (95% CI)	PR[a] (95% CI)	PR[a] (95% CI)
Nicotine dependence					
Low	450	Ref.	Ref.	Ref.	Ref.
Moderate	338	0.96 (0.72-1.28)	0.89 (0.74-1.07)	0.92 (0.78-1.09)	0.74 (0.61-0.89)
High	39	0.58 (0.29-1.18)	0.61 (0.37-1.02)	0.68 (0.46-1.02)	0.61 (0.37-1.00)
Quit attempts (last 18 months)					
Yes	132	1.18 (0.90-1.54)	1.00 (0.84-1.19)	1.04 (0.89-1.21)	1.16 (0.96-1.40)
No	735	Ref.	Ref.	Ref.	Ref.
Belief about the harmfulness of SHS to others					
Agree	833	Ref.	Ref.	Ref.	Ref.
Neither agree nor disagree	121	0.54 (0.33-0.89)	0.79 (0.57-1.11)	0.84 (0.61-1.14)	0.62 (0.43-0.89)
Disagree	39	0.42 (0.19-0.91)	0.72 (0.46-1.12)	0.78 (0.55-1.09)	0.69 (0.43-1.12)

CI: confidence interval; PR: prevalence ratio; SHS: second-hand smoke.

[a]Prevalence ratios and 95% confidence intervals were estimated using Poisson regression models with robust variance adjusted for age, sex, and educational level.

STROBE Statement—Checklist of items that should be included in reports of ***cross-sectional studies***

	Item No	Recommendation	Page No
**Title and abstract**	1	(*a*) Indicate the study's design with a commonly used term in the title or the abstract	1
		(*b*) Provide in the abstract an informative and balanced summary of what was done and what was found	1
**Introduction**			
Background/rationale	2	Explain the scientific background and rationale for the investigation being reported	1-2
Objectives	3	State specific objectives, including any prespecified hypotheses	2
**Methods**			
Study design	4	Present key elements of study design early in the paper	2
Setting	5	Describe the setting, locations, and relevant dates, including periods of recruitment, exposure, follow-up, and data collection	2
Participants	6	(*a*) Give the eligibility criteria, and the sources and methods of selection of participants	2
Variables	7	Clearly define all outcomes, exposures, predictors, potential confounders, and effect modifiers. Give diagnostic criteria, if applicable	2-3
Data sources/ measurement	8*	For each variable of interest, give sources of data and details of methods of assessment (measurement). Describe comparability of assessment methods if there is more than one group	*2-3*
Bias	9	Describe any efforts to address potential sources of bias	2-3
Study size	10	Explain how the study size was arrived at	2
Quantitative variables	11	Explain how quantitative variables were handled in the analyses. If applicable, describe which groupings were chosen and why	2-3
Statistical methods	12	(*a*) Describe all statistical methods, including those used to control for confounding	3
		(*b*) Describe any methods used to examine subgroups and interactions	3
		(*c*) Explain how missing data were addressed	3

		(*d*) If applicable, describe analytical methods taking account of sampling strategy	NA
		(*e*) Describe any sensitivity analyses	NA
**Results**			
Participants	13*	(a) Report numbers of individuals at each stage of study—eg numbers potentially eligible, examined for eligibility, confirmed eligible, included in the study, completing follow-up, and analysed	2
		(b) Give reasons for non-participation at each stage	NA
		(c) Consider use of a flow diagram	--
Descriptive data	14*	(a) Give characteristics of study participants (eg demographic, clinical, social) and information on exposures and potential confounders	4-9
		(b) Indicate number of participants with missing data for each variable of interest	3-S1
Outcome data	15*	Report numbers of outcome events or summary measures	4-6, 8-9, S2-S7
Main results	16	(*a*) Give unadjusted estimates and, if applicable, confounder-adjusted estimates and their precision (eg, 95% confidence interval). Make clear which confounders were adjusted for and why they were included	4-9, S2-S7
		(*b*) Report category boundaries when continuous variables were categorized	3
		(*c*) If relevant, consider translating estimates of relative risk into absolute risk for a meaningful time period	--
Other analyses	17	Report other analyses done—eg analyses of subgroups and interactions, and sensitivity analyses	--
**Discussion**			
Key results	18	Summarise key results with reference to study objectives	9
Limitations	19	Discuss limitations of the study, taking into account sources of potential bias or imprecision. Discuss both direction and magnitude of any potential bias	11
Interpretation	20	Give a cautious overall interpretation of results considering objectives, limitations, multiplicity of analyses, results from similar studies, and other relevant evidence	9-11
Generalisability	21	Discuss the generalisability (external validity) of the study results	11

**Other information**			
Funding	22	Give the source of funding and the role of the funders for the present study and, if applicable, for the original study on which the present article is based	13

*Give information separately for exposed and unexposed groups.

Note: An Explanation and Elaboration article discusses each checklist item and gives methodological background and published examples of transparent reporting. The STROBE checklist is best used in conjunction with this article (freely available on the Web sites of PLoS Medicine at http://www.plosmedicine.org/, Annals of Internal Medicine at http://www.annals.org/, and Epidemiology at http://www.epidem.com/). Information on the STROBE Initiative is available at www.strobe-statement.org.

© 2024 Fu M. et al.
